# Is it possible for people to develop a sense of empathy toward humanoid robots and establish meaningful relationships with them?

**DOI:** 10.3389/fpsyg.2024.1391832

**Published:** 2024-08-12

**Authors:** Elena Morgante, Carla Susinna, Laura Culicetto, Angelo Quartarone, Viviana Lo Buono

**Affiliations:** IRCCS Centro Neurolesi Bonino Pulejo, Messina, Italy

**Keywords:** empathy, human–robot interaction, humanoid robots, social robots, rehabilitation

## Abstract

**Introduction:**

Empathy can be described as the ability to adopt another person’s perspective and comprehend, feel, share, and respond to their emotional experiences. Empathy plays an important role in these relationships and is constructed in human–robot interaction (HRI). This systematic review focuses on studies investigating human empathy toward robots. We intend to define empathy as the cognitive capacity of humans to perceive robots as equipped with emotional and psychological states.

**Methods:**

We conducted a systematic search of peer-reviewed articles using the Preferred Reporting Items for Systematic Reviews and Meta-Analyses guidelines. We searched Scopus, PubMed, Web of Science, and Embase databases. All articles were reviewed based on the titles, abstracts, and full texts by two investigators (EM and CS) who independently performed data collection. The researchers read the full-text articles deemed suitable for the study, and in cases of disagreement regarding the inclusion and exclusion criteria, the final decision was made by a third researcher (VLB).

**Results:**

The electronic search identified 484 articles. After reading the full texts of the selected publications and applying the predefined inclusion criteria, we selected 11 articles that met our inclusion criteria. Robots that could identify and respond appropriately to the emotional states of humans seemed to evoke empathy. In addition, empathy tended to grow more when the robots exhibited anthropomorphic traits.

**Discussion:**

Humanoid robots can be programmed to understand and react to human emotions and simulate empathetic responses; however, they are not endowed with the same innate capacity for empathy as humans.

## 1 Introduction

Empathy is a multidimensional construct used to describe the sharing of another person’s feelings and the ability to identify with others and grasp their subjective experiences (Airenti, 2015). It covers a spectrum of phenomena, ranging from experiencing feelings of concern for others to feeling within oneself the feelings of others. This ability is a complex phenomenon that includes an affective component understood as the capacity to share the emotional status of other subjects, and a cognitive dimension that implies the ability to rationally understand the thoughts, feelings, and perspectives of others (Decety and Jackson, 2004; Eisenberg and Eggum, 2009; Decety and Ickes, 2011).

In other words, emotional empathy enables individuals to be influenced by the emotions of others, aiding in the recognition of one’s own and the interlocutor’s emotions, which allows them to create a mental representation of the thoughts and emotional states of their interlocutors (Leite et al., 2013). Empathy is an extremely adaptable and versatile process that permits social behavior in a variety of settings. Although it can be considered a specific feature of humans, prosocial actions brought about by empathy can occasionally be constrained by external circumstances. Hoffman (2001) showed that constraints on empathy stem from two primary factors: empathic over-arousal and interpersonal dynamics between the subject and the target of empathy. Empathic over-arousal materializes if indications of distress are exceptionally strong; in this case, the empathic concern shifts to a state of personal distress. Moreover, the nature of the relationship between the observer and the object of empathy significantly shapes the form of the prosocial actions undertaken by the observer. For instance, people are more likely to empathize with friends and relatives than strangers (Krebs, 1970). Empathic responses can be modulated by personal characteristics or situational contexts (De Vignemont and Singer, 2006).

At the neural level, studies on empathy-mediated processes have demonstrated the important role of networks involved in action simulation and mentalizing, depending on the information available in the environment. This neural network of empathy includes the anterior insula, somatosensory cortex, periaqueductal gray, and anterior cingulate cortex (Engen and Singer, 2013).

In recent years, neuroscientific approaches have increased the study of different forms of empathy in human–robot interaction (HRI) (Tapus et al., 2007; Riek et al., 2010). This field is expanding rapidly as robots become increasingly adept at sophisticated social skills (Vollmer et al., 2018). Humanoid robots have sociable abilities and the capacity to interact with humans to understand verbal and non-verbal communication, such as postures and gestures (Alves-Oliveira et al., 2019).

Humanoid robots can influence users’ emotional states and perceptions of social interactions (Saunderson and Nejat, 2019). Studies have explored how people attribute intentions, personality, and emotional meaning to robots, thus helping establish guidelines for designing more humane and engaging robotic interfaces. Using neuroimaging techniques, it is demonstrated that observation of human movements and observation of robotic movements activate the same brain areas, indicating that the anthropomorphic qualities of robots can elicit empathic responses in humans (Gazzola et al., 2007). This emphasizes the role of the mirror neuron system in regulating human empathy and imaginative processes. Mirror neurons facilitate not only the reproduction of observed actions but also emotional resonance with others. This system responds not only to human actions but also activates in response to actions performed by a robot (Iacoboni, 2009).

The robots understand human intentions using the properties of the mirror neuron system, and they may be able to more accurately anticipate human actions and respond to it more precisely (Han and Kim, 2010).

Empathy is viewed as an active body of ongoing emotional and cognitive exchanges rather than a singular phenomenon of emotional mirroring to develop a relationship between individuals and other agents over time.

Research on virtual humans and robots referred to as “advanced intelligent systems” when combined explores one of two main perspectives: (1) how humans empathize with advanced intelligent systems or (2) the impact of a robot’s empathetic behavior on humans.

The first viewpoint looks at how humans emotionally engage with robots that have human-like characteristics, and it does not necessarily need robots to be empathic. As for the second viewpoint, many academics have looked at different methods and algorithms to give robots empathy so they can recognize and respond to humans’ emotional states (Birmingham et al., 2022).

This bidirectional empathy can strengthen the bonds between humans and robots and improve the quality of interaction and trust.

This systematic review is focused on studies that investigated empathy in the HRI.

## 2 Materials and methods

### 2.1 Search strategy

We conducted a systematic review to investigate the construct of empathy in HRI. A literature review was performed in accordance with the Preferred Reporting Items for Systematic Reviews and Meta-Analyses (PRISMA) guidelines by searching PubMed, Web of Science, and Embase. We considered articles published between 2004 and 2023. The following key terms were used: (‘empathy’[MeSH Terms] OR ‘empathy’[All Fields]) AND (‘humans’[All Fields] OR ‘humans’[MeSH Terms] OR ‘humans’[All Fields] OR ‘human’[All Fields]) AND (‘robot’[All Fields] OR ‘robot s’[All Fields] OR ‘robotically’[All Fields] OR ‘robotics’[MeSH Terms] OR ‘robotics’[All Fields] OR ‘robotic’[All Fields] OR ‘robotization’[All Fields] OR ‘robotized’[All Fields] OR ‘robots’[All Fields]) AND (fha[Filter]). Only English texts were considered.

All articles were reviewed based on titles, abstracts, and full texts by two investigators (EM and CS) who independently performed data collection to reduce the risk of bias (i.e., bias of missing results, publication bias, time lag bias, and language bias). The researchers read the full-text articles deemed suitable for the study, and in cases of disagreement regarding the inclusion and exclusion criteria, the final decision was made by a third researcher (VLB). The list of articles was then refined for relevance, revised, and summarized, with the key topics identified from the summary based on the inclusion and exclusion criteria.

The inclusion criteria were as follows: (i) studies on the population of healthy adults and (ii) studies that included a psychometric assessment of empathy.

The exclusion criteria were as follows: (i) studies involving children and (ii) case reports and reviews.

### 2.2 Data extraction and analysis

Following the full-text selection, data extraction from the included studies was summarized in a table (Microsoft Excel—Version 2021). The summarized data included the assigned ID number, study title, year of publication, first author, study aims and design, study duration, method and setting of recruitment, inclusion and exclusion criteria, informed consent, conflicts of interest and funding, type of intervention and control, number of participants, baseline characteristics, type of outcome, time points for assessment, results, and key conclusions.

The agreement between the two reviewers (CS and EM) was assessed using the kappa statistic. The kappa score, with an accepted threshold for substantial agreement set at >0.61, reflected excellent concordance between the reviewers. This criterion ensured a robust evaluation of inter-rater reliability, emphasizing the achievement of a substantial level of agreement in the data extraction process.

## 3 Results

**Figure 1:**
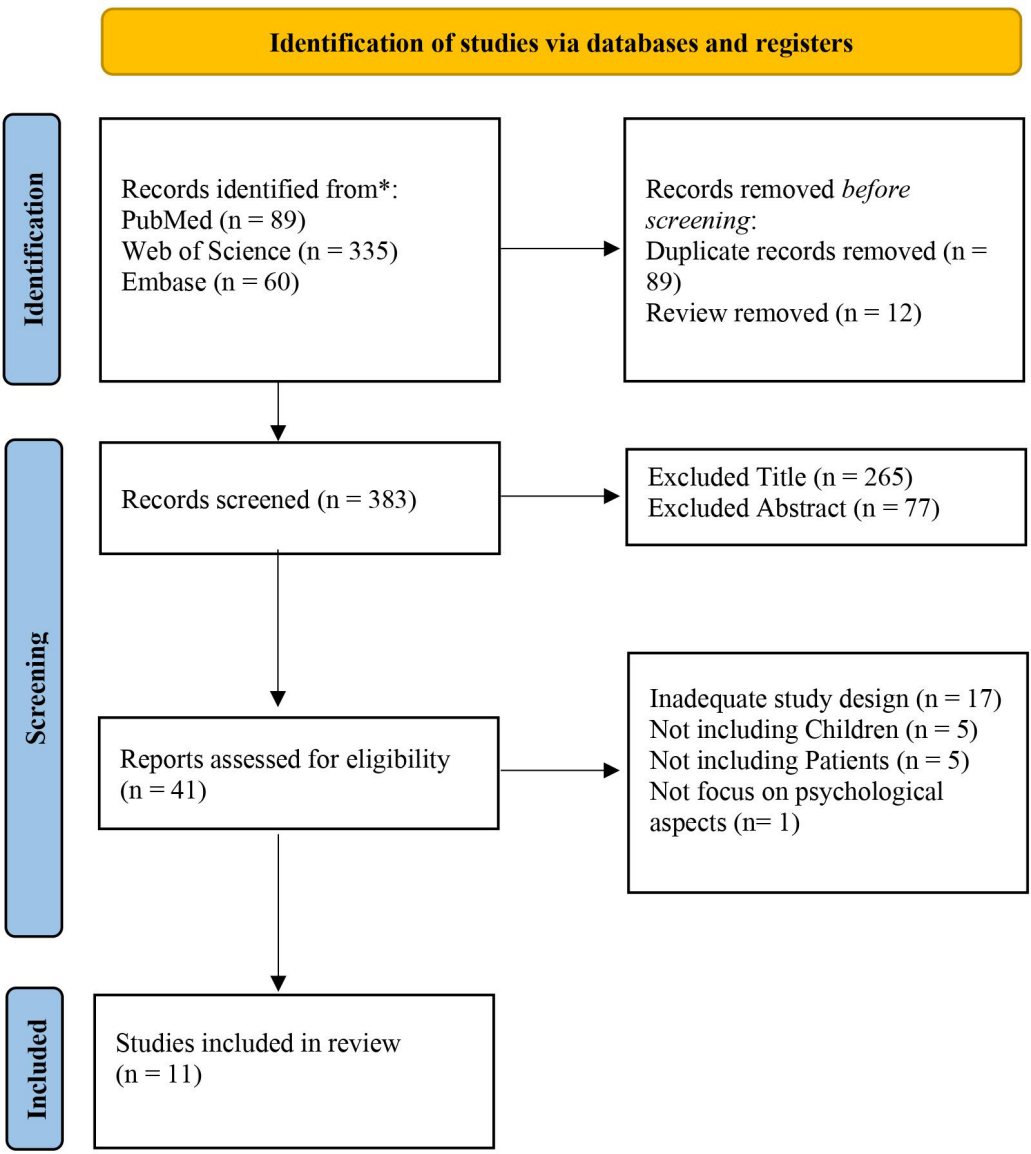
Search and selection of eligible articles.

A total of 484 articles were identified, including 89 from PubMed, 335 from Web of Science, and 60 from Embase. All articles were evaluated based on title, abstract, full text, and topic specificity. Only 11 studies met the inclusion criteria (Table 1; Figure 1).

**Table 1:** Studies assessing empathy.

Study	Aim	Sample (*N*)	Empathy assessment	Robotic agent	Outcomes
Empathic robot					
Birmingham et al. (2022)	Examining how viewers perceive cognitive and affective empathetic statements from a robot in response to human disclosure	111 Healthy Subjects	RoPE scale, modified from a first-person questionnaire to a third-person questionnaire	Empathic Agent	The participants rated the affective statements higher than the cognitive ones
Mollahosseini et al. (2018)	Assessing automated FER accuracy on robots interacting with humans, along with task engagement, empathy, and likability	16 Healthy Subjects	Designed Questionnaire	Ryan Companionbot	Participants rated the empathic robot higher in empathy and likability compared to non-empathic robot
Leite et al. (2013)	Assessing whether empathetic artificial companions enhance user relationships	40 Healthy Subjects	Designed Questionnaire	iCat	Participants rated the supportive robot higher in companionship, alliance, and self-validation
Empathic responses of humans					
Tsumura and Yamada (2022)	The text explores whether human empathy varies based on task difficulty and content	578 Healthy Subjects	12-item questionnaire modified from the IRI	Empathic Agent	Higher task difficulty promoted human affective empathy
Konijn and Hoorn (2020)	The significance of facial articulacy and emotions in optimizing human–robot communication	265 Healthy Subjects	Designed Questionnaire	Robot Alice and robot Nao/Zora	Humans showed less empathic and emotional responsiveness toward robots compared to humans
García-Corretjer et al. (2023)	Active collaboration enhances meaningful empathy between humans and robots	18 Healthy Subjects	Toronto Empathy Questionnaire (TEQ)	Robot Robobo	Participants trusted the robot’s suggestions amid uncertainty, demonstrating teamwork attitudes
Erel et al. (2022)	Examining whether non-humanoid robot gestures boost emotional support in human–human interaction	64 Healthy Subjects	Verbal Empathy	Non-humanoid robotic object	Robots performing empathetic gestures improve human emotional support interaction
Spaccatini et al. (2023)	Assessing how attributing a specific mind to a social robot affects empathy toward individuals in distress	269 Healthy Subjects	Online questionnaire on Qualtrics	Social Robot’s Anthropomorphism, Chatbot	The level of anthropomorphization of robots produces empathy in interaction with humans
Moon et al. (2021)	Studying non-verbal cues’ impact and mediation structure in human–robot interaction	48 Healthy Subjects	Designed Questionnaire	Social Robot ‘Hubot’	A non-verbal cue has an outweighing effect on empathy in HRI
Frederiksen et al. (2022)	Studying how a robot’s emotional story affects empathy in humans	220 Healthy Subjects	Designed Questionnaire	Kuri robot	Sad narrative increased participants’ empathy and willingness to help the robot
Corretjer et al. (2020)	To develop empathy through fun collaboration scenario in which a user and a social robot work together	10 Healthy Subjects	Designed Questionnaire	Robot Robobo	Developing empathy through engaging collaboration scenarios with a social robot

IRI, interpersonal reactivity index; RoPE, robot’s perceived empathy; FER, facial expression recognition.

Recognizing or anticipating how people will react to robots and how well robots will respond to humans may depend on an understanding of human empathy toward them.

Two distinct research areas address the topic of empathy in social robots. In the first, the human interlocutor is the observer of the robot, and the robot is the target of human empathy. In the second area investigated, the robot is the observer of the human and is designed to exhibit empathy to the human. Thus, in the selected studies, we found two empathy-based HRI design orientations: the expression of empathy and the induction of empathy. The expression of empathy means that humans feel that the social robot is empathizing with their emotions. Relative to empathy induction, the social robot expresses its emotions in advance, through which the interacting human feels empathy. In recent years, significant progress has been made in both areas.

### 3.1 Empathic encoded robot response

In a study by Birmingham et al. (2022), a robot that used affective empathic statements was perceived as having more empathy in comparison with a robot programmed to manifest cognitive empathy. To evaluate the interaction, participants completed a short survey after watching two demonstration videos of each condition. The study analyzed the relationship between the participants’ attitudes toward the robots, their assessment of how genuine they felt the interaction was, and their assessment of the robot’s empathy in each condition. Furthermore, the relevant finding concerns the participants’ belief that the interaction between the robot and the actors was credible, natural, and genuine. A few studies have focused on the human characteristics of human robots, such as facial expressions, which play an important role in social interactions and communication processes. In detail, Mollahosseini et al. (2018) studied the benefits of using an automatic facial expression recognition system in the spoken dialog of a social robot and how the robot’s sympathy and empathy would be affected by the accuracy of the system. In the experimental condition, the robot empathizes with the user through a series of predefined conversations. The results of the study indicate that the incorporation of an automatic facial expression recognition system allowed subjects to perceive the robot as more empathetic than in the other conditions. In a study by Leite et al. (2013), two players engaged in a chess game were accompanied by an autonomous robot expressing empathy. In this way, the robot acted as a social companion. In this study, the empathic behaviors reported in the literature were modeled in a social robot capable of inferring certain affective states of the human subject, reacting emotionally to these states, and commenting appropriately on a chess game. The results indicate that individuals toward whom the robot behaved empathetically perceived the robot as friendlier, which continues to support the hypothesis that empathy plays a key role in HRI. These findings serve to support investigations concerning HRI focusing on human emotions and the development of robots that are perceived as appropriately empathic and that can tailor their empathic responses to users.

### 3.2 Robot-dependent empathic human response

Moon et al. (2021) studied empathy induction, which outlined the appropriate emotional expressions for a social robot to elicit empathy-based behavior. Like human–human interactions, non-verbal cues have been found to significantly influence empathy and induced behavior when people interact with robots. Specifically, the results showed that non-verbal cues conveyed a negative emotion, appropriate to the situation; this had a decisive effect on perceived emotion, empathy, and behavior induction. It has also been shown that a robot’s affective narrative can also influence its ability to elicit empathy in human subjects. In the study by Frederiksen et al. (2022), the authors explore the stimulation of empathy by investigating interaction scenarios involving a robot that uses affective narratives to generate compassion in subjects, while failing to complete the task. Therefore, this study explores the relationship between the type of narrative conveyed by the robot (funny, sad, and neutral) and the robot’s ability to elicit empathy in interactions with human observers. The results demonstrate that the type of narrative approach of the robot was able to influence the level of empathy created during an interaction. Konijn and Hoorn (2020) compared the facial articulacy of humanoid robots to a human in affecting users’ emotional responsiveness, showing that detailed facial articulacy does make a difference. The results of the study showed that robots can arouse empathic reactions in humans; when these reactions are greater, the robot’s facial expression will be more complex. The expressiveness of the robot has an important communicative function and makes it usable in contexts such as healthcare and education, allowing users to affectively relate to the robot at a level appropriate to the task or objective. Corretjer et al. (2020) focused on the study of quantitative indicators of early empathy realization in a challenging scenario, highlighting how participation in a collaborative activity (solving a maze) between humans and robots influenced the development of empathy. In a subsequent study (Corretjer et al., 2020), they assessed empathy, using indicators such as affective attachment, trust, and expectation regulation. Through the development of these aspects in an atmosphere that is supportive, the participants in the study engaged in mutual understanding, listening, reflecting, and performing. Although the robots did not have anthropomorphic characteristics, the participants managed to establish a collaborative and empathetic relationship with them with the aim of achieving a common goal. Tsumura and Yamada (2022), in an experimental condition, studied the conditions required to develop empathy toward anthropomorphic agents. The findings demonstrated that greater task difficulty, independent of task content, increased human empathy toward robots. Spaccatini et al. (2023) examined the potential impact of anthropomorphized robots on human social perceptions. The authors induced anthropomorphization of social robots by manipulating the level of anthropomorphism of their appearance and behavior. The results demonstrated that anthropomorphic social robots were associated with higher levels of experience and agency. Furthermore, the type of mind attributed to the anthropomorphic social robot influences the empathy perceived by the human. Erel et al. (2022) have shown that the non-verbal gestures of a non-humanoid robot can increase emotional support in human–human interactions. This indicates that a robot even without anthropomorphic features can improve the way humans interact.

## 4 Discussion

Many studies on people’s empathy for robots have been published in the last few years, but there are also fundamental questions concerning the correct use of the term empathy (Niculescu et al., 2013; Darling et al., 2015; Seo et al., 2015). Generally, empathy can be described as the ability to comprehend and experience another person’s feelings and experiences and is a crucial component of human social interaction that promotes the growth of affection and social bonds (Anderson and Keltner, 2002). When considering humanoid robots, one may wonder whether people can develop empathy for a device (Malinowska, 2021).

The phenomenon of humans’ empathy toward robots has garnered significant attention in the field of HRI and is in some ways a controversial topic. As reported in numerous studies, empathy in HRI is bidirectional. On the one hand, humans can feel empathy toward robots; on the other, robots, with the progress of technology, are designed to be empathetic in interactions with humans. It is possible to feel empathy toward robots, especially when the latter possess human characteristics, are anthropomorphized (Breazeal, 2003; Paiva et al., 2017), and adopt human-like attitudes. When robots exhibit human-like facial expressions, gestures, or voices, people tend to perceive them as more relatable and emotionally expressive, which can trigger empathetic reactions (Riek et al., 2010).

Social robots with human-like features can affect how people feel about them, which in turn can impact the robots’ ability to convey emotions (Spaccatini et al., 2023). The modulation of voice tone has also been shown to be effective in promoting empathic processes (James et al., 2018). In addition, robots designed with expressive faces that can mimic sadness, happiness, or surprise are more likely to elicit empathetic responses from humans (Leite et al., 2013). Humans tend to see robots with human-like characteristics as more than just machines, attributing them with a sense of liveliness and even emotional capabilities. This can lead people to perceive anthropomorphic robots as companions, promoting acceptance and trust between humans (Zoghbi et al., 2009). Consequently, people are more likely to interpret the emotions expressed by such robots as genuine, which can facilitate emotional connections in human–robot interaction (Bartneck et al., 2007).

It was also examined how mirroring facial expressions could improve empathy in HRI. Robots capable of reproducing human facial expressions seem to significantly improve empathic engagement (Gonsior et al., 2011).

However, while giving robots human-like features can enhance their ability to express emotions and help people understand those emotions, it can also lead to a phenomenon known as the “Uncanny Valley (Mori, 2005; Misselhorn, 2009).” This effect describes a decrease in human empathy toward robots and an increase in discomfort as robots become more similar to humans (Mori et al., 2012). Based on several studies, it has been discovered that this effect occurs in environments with a high level of anthropomorphism and various sensory stimuli, including auditory, visual, and tactile cues (Nomura and Kanda, 2016). As a result, individuals may develop incorrect expectations of the robot’s cognitive and social abilities during prolonged interactions (Dautenhahn and Werry, 2004).

Several studies have shown that humans’ empathic involvement toward robots can extend to various situations, even those in which robots are perceived as being in difficulty or in need of help. In such a scenario, it has been seen that people may feel guilt or sadness when they observe a robot failing to complete a task or being mistreated (Darling et al., 2015).

The anthropomorphism of robots might influence the socio-cognitive processes of humans and the subsequent behavior of subjects toward them. In the study by Spatola and Wudarczyk (2021), a focus was placed on the emotional capabilities of the robot, pointing out that endowing robots with more complex emotions could lead to more anthropomorphic attributions toward them. Therefore, the perceived emotionality of robots, which is not limited to one type of emotion, could predict some of the characteristics of robot anthropomorphism (Schömbs et al., 2023).

This assumption is in line with the “Simulation Theory” which suggests that the way we understand the minds of others is through “simulating” the situation of another; therefore, it should be more immediate to empathize with the emotions and mental states of a robotic agent that has human characteristics (Mattiassi et al., 2021).

Even non-humanoid robots are capable of activating empathic responses; in fact, they can produce behaviors and responses that users perceive as social or emotional, promoting the development of empathy. For example, if a robot has been programmed to provide help or comfort, users are more likely to feel empathy toward it, regardless of its non-human physical characteristics (Erel et al., 2022).

As robotics and artificial intelligence continue to advance, integrating empathic capabilities into robots has emerged as a crucial area of research. Empathy, the ability to understand and respond to the emotions of others, is fundamental to human social interaction. Developing an empathetic robot, like any other robot, requires a clear definition of its purpose. Based on this purpose, designers can create interaction scenarios, and engineers can develop the robot’s software and hardware architecture (Park and Whang, 2022). Transferring this capability to robots promises to revolutionize various fields, including healthcare, education, and social care, by improving the quality and effectiveness of human–robot engagement (Johanson et al., 2023). For social agents to exhibit empathic behavior autonomously, they need to simulate the empathic processes; indeed, empathic robots are designed to recognize, interpret, and respond appropriately to human emotions, thus promoting more natural and meaningful interactions. These robots have the potential to provide companionship, support therapeutic interventions, and assist in the care of vulnerable populations, such as the elderly or people with special needs (Darling et al., 2015).

However, humanoid robots have significant limitations in terms of empathy. Humanoid robots cannot participate in social relationships, as they are defined in the empathic mode, because they do not satisfy the requirements of logical and purposeful subjectivity. A being with logical subjectivity can think, reason, and make decisions independently based on his or her own understanding. This concept means that, despite technological advances and progress in the empathic design of robots, they have limitations: Robots do not yet possess autonomous cognitive processes and therefore lack logic and intentionality. Their responses, while potentially sophisticated and human-like, are ultimately the result of programmed behaviors rather than authentic understanding or shared emotional experiences.

While they can recognize emotions such as sadness or anger, they have difficulty understanding the underlying causes or motivations. This is the prerequisite for true empathy, which requires not only recognizing emotions but also sharing and understanding the feelings involved. Most robots cannot feel real emotions on their own. They can simulate emotional reactions, but these are based on algorithms and data and not on real feelings (Chuah and Yu, 2021). Researchers on HRI have begun to investigate various aspects of empathy in robots. Understanding and feeling the emotions of another human person requires a high level of emotional awareness and understanding that current systems do not possess. Humanoid robots can be made to understand and respond to human emotions using pre-programmed algorithms and models. They can be programmed to simulate empathetic responses to some degree extent for certain applications in HRI and social robotics, but they do not have the same innate capacity for empathy as human people (Johanson et al., 2021). However, numerous studies in the field of HRI have shown that humans may empathize with and trust robots that can recognize their emotional states and respond appropriately to them (Kozima et al., 2004).

Regarding mental state perception/attribution, which is the cognitive ability to reflect on one’s own and others’ mental states such as beliefs, desires, feelings, and intentions by robots, studies have described contrasting results. While, on the one hand, people attribute the behavior of robots to underlying mental causes, on the other, they tend to deny that robots have a mind when explicitly requested to do so (Thellman et al., 2022).

While, on the one hand, people attribute the behavior of robots to underlying mental causes, on the other, they tend to deny that robots have a mind when explicitly requested to do so.

The bias of people to attribute mental states to robots is the outcome of multiple factors, including the motivation, behavior, appearance, and identity of robots. Endowing them with mental states helps to predict and explain their behavior, reduces uncertainty, and increases the sense of control in an interaction context (Epley et al., 2007; Eyssel et al., 2011; Levin et al., 2013; de Graaf and Malle, 2019). Indeed, it has also been found that people are more likely to attribute mental states to robots both when they are designed to exhibit socially interactive behavior and when they are endowed with a human-like appearance.

In most studies in the literature, it appears that the theory of mental state attribution is most often related to anthropomorphism, i.e., the attribution of mental abilities and human traits to non-human entities (Thellman et al., 2022).

Social robots are an increasingly important component of an improved social reality with relationships. Although true empathy in humanoid robots may still be a long way off, recent advances in social and developmental psychology, neuroscience, and virtual agent research have shown promising avenues for the development of empathic social robots (Guzzi et al., 2018). Seibt (2017) has classified different levels and degrees of sociality in human–robot interactions within the social interactions framework (SISI) and used the concept of ‘simulation’ to distinguish between full realization, partial realization, and different simulated forms of social processes, such as approximation, representation, imitation, mimicry, or replication. SISI can simulate some aspects of this complexity, but it cannot fully replicate the real-time dynamics and emotional subtleties of real human interactions (Seibt, 2017).

The main limitation of this review is the significant weakness in defining empathy, as it is not a directly observable construct but can only be inferred from behavior, and there is no clear definition or global agreement on how to measure empathic abilities in robots.

Human beings’ attributions of robots are related to dimensions of mental perception. These depend on both experience and behavior and suggest that the more mental state attribution capabilities are ascribed to robots, the more they are likely to be valued (Gray et al., 2007).

Furthermore, the overall quality of evidence was low and the selected studies differed greatly in their definitions, assessment tools, and outcome measures. Due to the lack of standardized protocols, a meta-analysis could not be conducted. Regarding the assessment of perceived empathy, the way humans empathize with robots can be measured by their behavior toward robots (Spatola and Wudarczyk, 2021). Empathic emotions can be expressed through facial expressions, bodily expressions, physiological reactions, and action tendencies, and then through explicit measures such as surveys (Carpinella et al., 2017), and currently also through neuroscientific measures (e.g., EEG, MRI, and fNIRS). Although various questionnaires are available to study empathy in humans, in particular Davis’ questionnaire (Davis, 1983), which is undoubtedly a benchmark for measuring individual differences in empathy, many researchers have developed their measures without relying exclusively on the currently existing instruments. The main controversy in assessment concerns the fact that to assess robot-induced empathy, one must rely on human subjects’ perception of empathic traits, which means that one must measure the degree of ‘perceived empathy’. The evaluation has a major impact on future developments and on whether more emphasis should be placed on certain algorithms or certain functional constructs rather than others. Therefore, evaluations also provide data that will influence the creation of new models for robot behavior, which in turn will affect the many different new applications.

The implications of empathy in HRI are manifold. Another important aspect is understanding why humans feel empathy toward robots as this influences the design and effectiveness of these interactions (Leite et al., 2014; Stock-Homburg, 2022). The goal of researchers must be to develop new design models to increase the emotional intelligence and social integration of robots and ultimately create more effective and realistic human–robot interactions (Damiano et al., 2015).

Improving these interactions must both increase the quality of the user experience and have beneficial therapeutic outcomes. Despite promising applications, the development of truly empathetic robots is fraught with complex challenges, including ethical implications. While empathy enhances human–robot interactions, it also raises ethical questions about the nature of these interactions and the potential for emotional manipulation (Coeckelbergh, 2010). To improve the utility and acceptance of robots in society, future perspectives must also consider these implications and ensure that robots are designed to promote positive and healthy human–robot interactions without exploiting human emotions (Zhou and Shi, 2011).

## Data availability statement

The data presented in the study are included in the article/supplementary material, further inquiries can be directed to the corresponding author/s.

## Author contributions

EM: Methodology, Writing – original draft. CS: Methodology, Writing – original draft. LC: Writing – review & editing. AQ: Supervision, Writing – review & editing. VL: Conceptualization, Methodology, Supervision, Writing – review & editing.
